# Editorial: Clinical Microbiology in Low Resource Settings

**DOI:** 10.3389/fmed.2020.00258

**Published:** 2020-06-10

**Authors:** Zisis Kozlakidis, Olivier Vandenberg, John Stelling

**Affiliations:** ^1^International Agency for Research on Cancer, World Health Organization, Lyon, France; ^2^Innovation and Business Development Unit, LHUB - ULB, Groupement Hospitalier Universitaire de Bruxelles (GHUB), Université Libre de Bruxelles, Brussels, Belgium; ^3^Centre for Environmental Health and Occupational Health, School of Public Health, Université Libre de Bruxelles (ULB), Brussels, Belgium; ^4^Division of Infection and Immunity, Faculty of Medical Sciences, University College London, London, United Kingdom; ^5^Microbiology Laboratory, Women's Hospital, Boston, MA, United States

**Keywords:** low resource, clinical, microbiology, antimicrobial resistance (AMR), surveillance

The field of clinical microbiology faces increased pressures in low and middle-income countries (LMICs) where the burden of infection is highest and health systems appear least able to respond to pressures, such as antimicrobial resistance (1). The current gaps in our knowledge relating to detection, characterization, effective treatment, and follow-up constrain national governments and international organizations in their efforts to detect evolving trends and emerging threats.

Good quality clinical microbiology, implemented at a population level through well-functioning reference laboratory networks is necessary for effective antimicrobial resistance surveillance and control for example, but low-resource settings face infrastructural, technical, and human resource challenges toward such an implementation. As is true for many topics, hospitals and communities in different LMIC settings have unique challenges and methods for overcoming those challenges fully or partially (2). The regional and national variations of clinical microbiology implementation in LMICs limit our understanding and the extent to which clinical microbiology results can inform national and international health policies. An important component and common limitation of health systems in LMICs are the human resources to do work, across all cadres of staff, including clinical, laboratory, managerial, policy-making, data analysis, and project management groups (3).

Microbiological expertise is particularly limited in the generation, sharing, systematic analysis, and dissemination of data in low-resource settings. Numerous agencies and initiatives are working to support the development of clinical microbiology capacity. However, the routine generation of a predictable, sustainable flow of reliable data will take time to establish and deliver clinical and public health impact (4). An integrated model, combining clinical, laboratory and demographic information remains perhaps a distant aspiration. As such the need to share knowledge and experiences is an effective approach to remove communication barriers and contribute to reducing the inequities in understanding disease burden worldwide.

The expectation for clinical microbiology laboratories in LMICs to be technology-ready and able to swiftly incorporate new technological solutions is currently difficult to materialize. New technologies, such as next-generation sequencing diagnostics, do have the potential to increase the surveillance granularity of microbial pathogens and eventually widen the opportunities for more effective healthcare implementation. They have also proven workable when operated in isolation. However, there are a number of foundational needs that still need to be addressed in LMIC settings. For example, the standardization or at least harmonization of protocols for existing methodologies across different geographies—even within the same country—remains a critical first step.

This themed special issue aims to explore the implementation of clinical microbiology in LMICs through comparative and evidence-based examples from across the world. The special themed issue incorporates critical, theoretically informed, and empirically grounded contributions translated into 2 reviews and 3 original research articles.

Jacobs et al. summarize the challenges and barriers to integrating clinical bacteriology in hospitals in low-resource settings, as well as the opportunities provided by recent capacity building efforts of national laboratory networks focused on vertical single-disease programs.

Ombelet et al. review the best practices of blood cultures in Low- and Middle-Income Countries, and present recommendations regarding appropriate blood culture sampling and processing in such settings.

These two reviews are followed by organism- and method-specific manuscripts, providing further such clinical evidence. Hadyeh et al. presents the molecular characterization of methicillin resistant *Staphylococcus aureus* in the West Bank-Palestine.

Mboera et al. explores the mortality patterns of toxoplasmosis and its comorbidities in Tanzania through a 10-year retrospective hospital-based survey, highlighting the acute need for further public health interventions.

Budayanti et al. identifies the quality thresholds of clinical sputum specimens as a predictor of isolated bacteria from patient with lower respiratory tract infections at a major Indonesian tertiary hospital.

As evidenced by the above, the slow yet gradual improvement of clinical microbiology in low-resource settings is expected to facilitate the future exploitation of routine patient care data for surveillance, antibiotic stewardship, and infection prevention and control. However, for this to take place, a consistent effort needs to be made toward that goal. A major leap forward can be achieved if challenges related to staff training and retention, funding, scale and the specific nature of clinical microbiology are prioritized. However, this would necessitate the synergistic efforts of multiple stakeholders—as has been demonstrated during the mobilization of resources and efforts for vertical programmes relating to HIV, TB, and malaria. Such plans require a good understanding of the healthcare challenges, so that any interventions can be inclusive of multiple facets (from diagnostic microbiology to business models) and hence become tailored for a successful implementation.

The future of the clinical microbiology laboratory in LMICs shall not be an “entry-level version” of its counterpart in high-resource settings. Conversely it will become a purpose-built, well-conceived, cost-effective and efficient laboratory ready to assume its place at the frontline of antimicrobial resistance containment; microbial pathogen detection, identification and treatment.

## Author Contributions

ZK, OV, and JS wrote and approved this editorial for publication.

## Conflict of Interest

The authors declare that the research was conducted in the absence of any commercial or financial relationships that could be construed as a potential conflict of interest. The handling editor declared a past co-authorship with two of the authors ZK and OV.
